# Movement of seed- and soil-applied fluopyram in soil columns

**DOI:** 10.21307/jofnem-2019-045

**Published:** 2019-07-31

**Authors:** Travis R. Faske, Katherine Brown

**Affiliations:** 1 Division of Agriculture, University of Arkansas, Lonoke Extension Center, Lonoke, AR 72086

**Keywords:** Abamectin, *Bacillus firmus*, Behavior, *Meloidogyne incognita*, Thiodicarb

## Abstract

The movement of seed- and soil-applied fluopyram was evaluated in soil columns. The nematicide was sampled at three soil depths and used in a nematode motility bioassay. Based on *Meloidogyne incognita* mortality, the downward movement of soil-applied fluopyram was affected by soil type and application method. No nematode-toxic levels of soil-applied fluopyram were detected past 5 cm depth in sandy loam soil compared to 10 cm depth in sandy soil. A slower rate of water infiltration had little impact on the movement of soil-applied fluopyram in sandy soil, but did affect the movement of soil-applied abamectin. In the seed-applied nematicide experiments, a greater effect on nematode mortality was observed at the 0 to 5 cm depth in sandy soil with fluopyram- than abamectin-treated cotton seed, whereas a similar effect was observed with soybean seed. No effect on nematode motility was observed with other seed-applied nematicides, thiodicarb, and *Bacillus firmus*. Overall, soil-applied fluopyram had a greater effect on *M. incognita* mortality at 10 cm depth in sandy soil than seed-applied fluopyram. These data provide a better understanding as to the movement of fluopyram as affected by soil type, water infiltration rates, and application methods.

The southern root-knot nematode, *Meloidogyne incognita*, is among the most important plant-parasitic nematodes affecting upland cotton (*Gossypium hirsutum*) and soybean (*Glycine max*) production in the southern USA (Starr et al., 2007; Koenning, 2015; Weaver, 2015). During the 2017 cropping season yield loss estimates by the southern root-knot nematode in cotton were 2.0% or 629,000 bales across the US Cotton Belt (Lawrence et al., 2018), while losses by *Meloidogyne* spp. in soybean were 1.2% or 22 million bushels of grain across the southern USA (Allen et al., 2018).

Nematicides continue to be an important part of an integrated system to manage root-knot nematodes in cotton and soybean. They are most often utilized when *M. incognita*-resistant cultivars or non-host crop options are lacking. The presence of multiple species of economically important nematodes in a field may also require the use of a nematicide to limit yield losses. Fumigant nematicides are highly effective, but may require additional equipment and have higher production cost than nonfumigants. Nonfumigant nematicides applied to seed and soil are widely used in row crop agriculture. During the past 20 years there has been a general trend to market nonfumigant nematicides that have a lower risk to human safety and impact to non-target organisms. Currently, one such nematicide being evaluated for use in cotton and soybean is fluopyram.

Fluopyram is classified as a succinate dehydrogenase inhibitor fungicide that is used extensively as a seed- and soil-applied nematicide in cotton and soybean. Fluopyram has been reported to be toxic to several species of plant-parasitic and free-living nematodes (Faske and Hurd, 2015; Heiken, 2017; Beeman and Tylka, 2018). However, suppression of *M. incognita* infection on cotton and soybean has been somewhat variable in field trials (Hurd et al., 2017; Jackson et al., 2017; Faske et al., 2018). Although the efficacy of fluopyram has been evaluated, the dynamics of the compound in soil as related to nematode control is unknown.

The objectives of this study were to evaluate the effect of soil texture and soil water infiltration rate on the movement of soil-applied fluopyram, and to evaluate the movement of fluopyram from treated cotton and soybean seed.

## Materials and methods

### Nematode inoculum

*Meloidogyne incognita* were isolated from field-grown cotton (*G. hirsutum*) and maintained on tomato (*Solanum lycopersicum* “Rutgers”). Eggs of *M. incognita* were collected from infested tomato roots with 0.5% NaOCl (Hussey and Barker, 1973) and second-stage juveniles (J2) were collected in a hatching chamber (Vrain, 1977). Only 24-hour-old J2 were used in this study.

### Cotton and soybean treated seed

Cotton cv. Stoneville ST 4747 GLB2 with commercially applied seed treatments were used in this study. All cotton seed were treated with a base fungicide treatment of metalaxyl + penflufen + prothioconazole + mycolbutanil (Allegiance^®^ FL + EverGol^®^ Prime + Proline^®^ 480 SC, Bayer CropScience, Research Triangle Park, NC and Spera^TM^ 240 FS, Nufarm Americas Inc., Alsip, IL) at 0.015+0.005+0.005+0.003 mg ai/seed, respectively. Seed-applied nematicide and insecticide treatments consisted of imidacloprid + thiodicarb (Aeris^®^ Seed-Applied Insecticide/Nematicide, Bayer CropScience, Research Triangle Park, NC) at 0.75 mg ai/seed, abamectin (Avicta^®^ 500 FS, Syngenta Crop Protection, Greensboro, NC) at 0.15 mg ai/seed + thiamethoxam (Cruiser^®^ 5 FS, Syngenta Crop Protection, Greensboro, NC) at 0.34 mg ai/seed, and fluopyram (COPeO^®^ Prime, Bayer CropScience, Research Triangle Park, NC) at 0.25 mg ai/seed + imidacloprid (Gaucho^®^ 600 FS, Bayer CropScience, Research Triangle Park, NC) at 0.375 mg ai/seed.

Soybean cv. Hornbeck HBK RY4721 with commercially applied seed treatments were used in this study. All soybean seed were treated with a base fungicide treatment of prothioconazole + penflufen + metalaxyl (EverGol^®^ Energy SB, Bayer CropScience, Research Triangle Park, NC) applied at 0.02 mg ai/seed. Seed-applied nematicides and insecticides consisted of clothianidin + *Bacillus firmus* I-1582 (Poncho^®^/VOTiVO^®^ Bayer CropScience, Research Triangle Park, NC,) at 0.13 mg ai/seed, abamectin (Avicta^®^ 500 FS) at 0.15 mg ai/seed + thiamethoxam (Cruiser^®^ 5 FS, Syngenta Crop Protection, Greensboro, NC ) at 0.12 mg ai/seed, and fluopyram (ILeVO^®^, Bayer CropScience, Research Triangle Park, NC ) at 0.15 mg ai/seed + imidacloprid (Gaucho^®^ 600 FS) at 0.12 mg ai/seed.

### Soil-applied nematicide experiments

Four experiments were conducted to evaluate the movement of fluopyram as a water dilution in soil columns with different soil types, water infiltration rates, and soil volume. Soil columns were constructed by filling a plastic tube (6-mm-diam.×160-mm-long) with <2.0-mm-diam. sterilized sandy soil (100% sand; pH 6.7; CEC 2.0 cmol+/kg) and wetted to field capacity by applying a total of 1.8 ml sterilized distilled water. To each column, 25.0 µg of agricultural grade fluopyram (Velum^®^ Prime, Bayer CropScience, Research Triangle Park, NC) in 100 µl of distilled water was applied on the soil surface and incubated overnight (16 hr) in a resealable plastic bag. The concentration of fluopyram used is estimated to be 10% of that used on cotton seed and the liquid formulation of fluopyram + imidacloprid used in cotton. Agricultural grade abamectin (Avicta^®^ 500 FS, Syngenta Crop Protection, Greensboro, NC) at 25.0 µg was used as the industry standard and distilled water as a negative control. Soil columns were drenched with 0.12 µl water/mm^3^ soil at a base rate of water infiltration of 20.0 mm/d on day two and incubated overnight in a resealable plastic bag. Plastic tubes were cut into 5-cm-long segments on day three and the soil within each segment dislodged into a 5-ml centrifuge tube (Eppendorf Ag, Hamburg, Germany) that contained 1 ml sterilized distilled water and vortexed for 30 sec. The supernatant was used immediately in a nematode motility bioassay. These bioassays were performed in 24-well Falcon tissue cultures plates (Corning Life Science, Tewksbury, MA). Each well received 500 µl of supernatant from a single centrifuge or conical tube, which contained 30 to 40 J2 in 500 µl of distilled water and incubated at 28°C for 24 hr. Second-stage juvenile motility was determined visually with an inverted compound microscope (Axio Vert.A1, Carl Zeiss Microscopy, Thornwood, NY). Nematodes were considered dead if they did not respond to being touched by a small probe and the percent of dead nematodes were recorded. Treatments were arranged in a completely randomized design (CRD) with three replications and the experiment was conducted three times.

To evaluate the effect of soil texture on fluopyram movement, plastic tubes (6-mm-diam.×160-mm-long) filled with <2.0-mm-diam. sterilized sandy loam soil (62% sand, 30% silt, and 7% clay; <1% organic matter; pH 6.0; CEC 11.3 cmol+/kg) were used in the second experiment. Methods used were the same as described in the first experiment with the exception of 1.9 ml water used to wet soil to field capacity and that 5-ml tubes were centrifuged at 25,000 RPM for 3 min to remove some silt and clay particles from the supernatant to better visualize nematodes in bioassay. Treatments were arranged in a CRD with three replications and each experiment was conducted three times.

The effect of a slower rate of water infiltration on fluopyram movement was investigated in the third experiment. Soil columns were constructed with sandy soil and nematicide treatments applied as described in experiment one. A slower rate of water infiltration was achieved by applying the same water volume (0.12 µl water/mm^3^ soil) in 20 µl aliquots most days over a 30-day period. Methods for supernatant collection and nematode bioassay were as described previously. Treatments were arranged in a CRD with three replications and each experiment was conducted three times.

The movement of soil-applied fluopyram was evaluated in a larger volume of soil in the fourth experiment. Soil columns were constructed by filling a plastic tube (12-mm-diam.×160-mm-long) with sandy soil (100% sand) and wetted to field capacity with 5 ml distilled water. Nematicide treatments and methods used were as described in experiment one. Soil columns were drenched with 0.12 µl water/mm^3^ soil at a base rate of water infiltration of 20.0 mm/d on day two and incubated overnight in a plastic bag. Plastic tubes were cut into 5-cm-long segment on day three and the soil within each segment was dislodged into a 15-ml conical tube that contained 5 ml sterilized distilled water and vortexed for 30 sec. Supernatant was used immediately in a nematode motility bioassay, described previously. Treatments were arranged in a CRD with three replications and the experiment was conducted twice.

### Seed-applied nematicide experiments

Two experiments were conducted to evaluate the movement of fluopyram from the seed coat of cotton and soybean seed. Soil columns (6-mm-diam.) were constructed with sandy soil as described previously. Fluopyram-treated cotton seeds were placed 2.0 cm below soil surface and incubated overnight in a resalable plastic bag. Other seed-applied nematicide treatments consisted of imidacloprid + thiodicarb and abamectin. Fungicide-treated cotton seed served as the negative control. Methods for water drench (base rate of water infiltration), supernatant collection, and nematode bioassay were as described previously for 6-mm-diam. soil column. Treatments were arranged in a CRD with three replications and the experiment was conducted twice.

In the second seed-applied nematicide experiment, fluopyram movement from soybean seed was investigated. Twelve-mm-diameter soil columns were constructed with sandy soil as described previously. The larger column was used to accommodate the larger soybean seed. Fluopyram-treated soybean seeds were placed 2.0 cm below soil surface and incubated overnight in a resalable plastic bag. Other seed-applied nematicide treatments consisted of clothianidin + *B. firmus* and abamectin. Fungicide-treated soybean seed served as the negative control. Methods for water drench (base rate of water infiltration), supernatant collection, and nematode bioassay were as described previously for 12-mm-diam. soil column. Treatments were arranged in a CRD with three replications and the experiment was conducted twice.

### Statistical analysis

Percent nematode mortality data were arcsine transformed (arcsine (sqrt(*x*))) to normalize for analysis and non-transformed data are reported. Data within each experiment were analyzed using a factorial ANOVA in the general linear mixed model procedure with experiment repetitions and treatment replications modeled as random variables, while nematicides and soil depths were modeled as fixed variables using SPSS 25.0 (SPSS Inc. Chicago, IL). Means were separated according to Tukey’s honest significant difference (HSD) test at *α* = 0.05.

## Results

**Figure 1:**
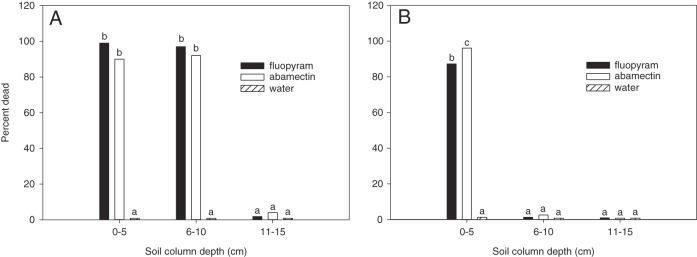
Percent dead *Meloidogyne incognita* after 24 hr exposure to nematicides detected at three depths in sand (A) and sandy loam (B) filled 6-mm-diameter soil columns drenched with a base rate of water infiltration. The base rate of water infiltration was 20.0 mm/d. Different letters over bars indicate significant differences at *α* = 0.05 according to Tukey’s HSD test.

There was no (*P* > 0.05) experiment by nematicide by soil depth interaction for any the soil-applied nematicide experiments. However, there was an interaction (*P* ≤ 0.001) between nematicide and soil depth for each experiment. In the first experiment with sandy soil and base rate of water infiltration, a similar effect of nematode motility was observed at 0 to 5 and 6 to 10 cm soil depth for abamectin and fluopyram with an average J2 mortality of 91 and 98%, respectively (Fig. 1A). No effect on J2 motility was observed beyond 10 cm soil depth for either nematicide.

In the second experiment with sandy loam soil and base rate of water infiltration, a greater (*P*≤0.05) effect on nematode activity was observed at 0 to 5 cm soil depth for abamectin at 96% mortality compared to 87% for fluopyram (Fig. 1B). There was no effect on J2 motility beyond 5 cm soil depth for either nematicide (Fig. 1B). Compared to the first experiment with sandy soil, the movement of both abamectin and fluopyram was limited in sandy loam soil.

**Figure 2:**
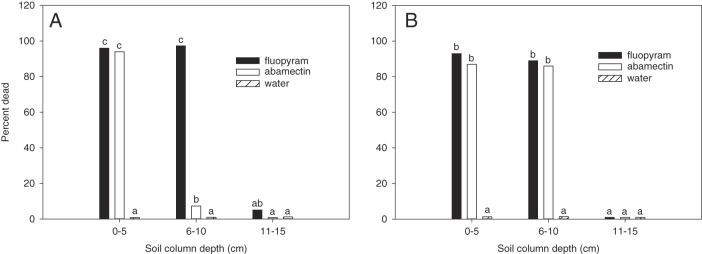
Percent dead *Meloidogyne incognita* after 24 hr exposure to nematicides detected at three depths in sand filled 6-mm-diameter soil column drenched with a slower rate of water infiltration (A) and a 12-mm-diameter soil column drenched with a base rate of water infiltration (B). The base rate was 20.0 mm/d whereas the slower rate was 0.7 mm/d over a 30-day period. Different letters over bars indicate significant differences at *α* = 0.05 according to Tukey’s HSD test.

In the third experiment with sandy soil and a slower rate of water infiltration, a similar effect on nematode activity was observed at 0 to 5 cm soil depth with 97 and 94% J2 mortality for fluopyram and abamectin, respectively (Fig. 2A). However, a greater (*P*≤0.05) effect on nematode activity was observed at 6 to 10 cm soil depth for fluopyram at 97% mortality compared to 7% for abamectin. No effect on nematode activity was observed beyond 10-cm soil depth for either nematicide.

In the fourth experiment with sandy soil, base rate of water infiltration, and a larger (12-mm-diam.) soil column, a similar effect on nematode activity were observed at 0 to 5 and 6 to 10 cm soil depth with an average J2 mortality of 87% for abamectin and 91% for fluopyram (Fig. 2B), while there was no effect on nematode activity beyond the 10 cm soil depth. Similar results were observed with that of the 6-mm-diam. soil column and base rate of water infiltration in the first experiment.

**Figure 3:**
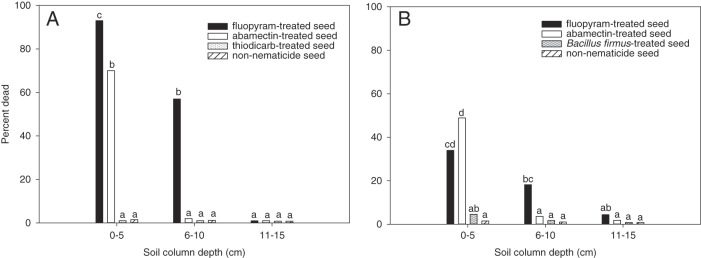
Percent dead *Meloidogyne incognita* after 24 hr exposure to nematicides from cotton (A) and soybean (B) treated seed detected at three depths in sand filled soil columns drenched with a base rate of water infiltration. The base rate of water infiltration was 20.0 mm/d. A 6-mm-diam. column was used for cotton seed, while a 12-mm-diam. column was used for soybean seed. Different letters over bars indicate significant differences at *α* = 0.05 according to Tukey’s HSD test.

In the seed-applied nematicide experiments, there was no (*P* > 0.05) experiment by nematicide by soil depth interaction for cotton or soybean seed; however, there was an interaction (*P*  0.001) between nematicides and soil depth for each experiment. A greater (*P*≤0.05) effect on nematode activity was observed for fluopyram detected at 0 to 5 cm soil depth than abamectin, thiodicarb, and the control cotton seed (Fig. 3A). Similarly, a greater (*P*≤0.05) effect on J2 activity was observed with fluopyram detected at 6 to 10 cm depth with 57% dead compared to <2% for abamectin, thiodicarb, and the non-nematicide treated seed. Other than fluopyram, only abamectin affected J2 motility at 0 to 5 cm depth with 70% dead. Thiodicarb had no effect on nematode motility. No effect on nematode motility was observed beyond 10 cm soil depth for any seed-applied nematicide on cotton.

Nematicide movement from soybean seed was similar for abamectin and fluopyram at 0 to 5 cm soil depth as indicated by 49 and 34% J2 mortality, respectively (Fig. 3B). Both nematicides had a greater (*P*≤0.05) effect on J2 mortality than *Bacillus firmus*, which had no effect on nematode motility. Fluopyram had a greater (*P*≤0.05) effect on nematode motility at 6 to 10 cm column depth than the other seed-applied nematicides. No effect on nematode activity was observed beyond 10 cm soil depth for any seed-applied nematicide on soybean.

## Discussion

These data indicate that the downward movement of the nonfumigant nematicide, fluopyram, is affected by soil type and application method. This may account for difference in the field efficacy of fluopyram that have been reported. Most of the fluopyram and abamectin in sandy loam soil remained in the upper 5 cm soil depth, while in sandy soil moved slightly deeper, but remained in the upper 10 cm soil depth. Similar results were reported with avermectin B_1a_, one of the main (80%) components of abamectin, where 90% of the abamectin remained in the upper 6 cm of a soil column filled with sandy loam soil (Gruber et al., 1990). The movement of other nonfumigant nematicides and insecticides were reported to be limited in soils with small pore spaces and fine particle sizes (Harris, 1972; Bromilow, 1973; Whitehead, 1973). Thus, fluopyram may be less effective in finer textured soils.

A slower rate of water infiltration had less impact on the movement of fluopyram than on abamectin. Strongly adsorptive compounds such as abamectin are less mobile, while weakly adsorbed compounds are more easily distributed in soil by water infiltration (Smelt and Leistra, 1992). Abamectin has very low mobility, very low water solubility (0.0078 mg/L) and a high soil adsorption coefficient (4,000-5,000 ml/g), while fluopyram has moderate mobility, low water solubility (16.0 mg/L) with a lower soil adsorption coefficient (233-440 ml/g) (Wislocki et al., 1989; Wauchope et al., 1992; ESFA, 2013). It is generally accepted that some water infiltration is necessary to distribute nonfumigant nematicides beyond the point of application (Bromilow, 1973). In preliminary experiments (data not shown), water was necessary to distribute seed- and soil-applied fluopyram beyond 5 cm soil depth in sandy soil. Based on nematode motility neither fluopyram nor abamectin was detected at nematode-toxic levels beyond 10 cm depth in sandy soil. Although the mobility of abamectin is low it is more toxic to *M. incognita* than fluopyram (Faske and Starr, 2006; Faske and Hurd, 2015), therefore, only a small amount was needed to cause a similar incidence of *M. incognita* mortality. The limited downward movement of abamectin in this study is comparable to other soil column and field studies (Gruber et al., 1990; Gannon et al., 2017). Though the downward movement of fluopyram was limited to 10 cm soil depth, the concentration detected would inhibit J2 infection of tomato (Faske and Hurd, 2015).

The concentration of fluopyram and abamectin detected from treated cotton and soybean seed was greater in the upper 5 cm soil depth with less detected at other soil depths. In general, seed-applied abamectin and fluopyram had a lower effect on *M. incognita* mortality compared to soil-applied at the same soil depth in sandy soil. For example, 70% J2 mortality was observed with fluopyram-treated cotton seed at 6 to 10 cm soil depth compared to 97% for soil-applied fluopyram. Given that cotton seed was treated with 250 µg fluopyram compared to 25 µg used in the soil-applied experiments, suggest that the majority of the fluopyram remained on the cotton seed coat. The more rapid distribution of soil-applied fluopyram as a water dilution in soil may provide better root protection from plant-parasitic nematodes than seed-applied fluopyram. Fluopyram applied in-furrow was reported to provide better seedling root protection from *M. incognita* compared to seed-applied fluopyram in field trials across the US Cotton Belt (Lawrence et al., 2016; Faske et al., 2017, 2018).

In comparison between seed types, in general, the concentration of fluopyram and abamectin detected at 0 to 5 and 6 to 10 cm soil depth from cotton seed contributed to a greater percentage of J2 mortality than that from soybean seed. Although more fluopyram was applied on cotton seed (250 µg/seed) than soybean (150 µg/seed), both seeds were treated with the same concentration of abamectin (150 µg/seed). Further, based on the known concentration response [*y* = 101.02/(1+exp(−(*x*−1.24)/1.93))] for *M. incognita* (Faske and Hurd, 2015) and J2 mortality after 24 hr exposure, the concentration of fluopyram detected in the upper 5 cm soil depth was estimated at 11.94 and 2.0 µg/ml for cotton and soybean seed, respectively. These concentrations would account for 4.8 and 1.3% of the fluopyram applied on the cotton and soybean seed coat, respectively. Thus, shortly after planting followed by a base rate of water infiltration only a small portion of the chemical was transferred from the seed coat into the soil water with some variation between cotton and soybean seed. In addition, as reported here and elsewhere, soil texture, soil structure (e.g. macropores), soil moisture, and soil water infiltration rate would impact the movement of seed-applied fluopyram (Bromilow, 1973; Smelt and Leistra, 1992; Flury, 1996; Rich et al., 2004; Nyczepir and Thomas, 2009; Wheeler et al., 2013).

Of the other seed-applied nematicides tested, none had an effect on nematode motility. Carbamate nematicides like thiodicarb have been reported to vary in their effect on *M. incognita* motility (Nordmeyer and Dickson, 1985). Thus, a longer exposure time may have been needed to visualize any effect on J2 motility with thiodicarb. Seed-applied *B. firmus* had no effect on *M. incognita* motility in this study. Secondary metabolites produced by *B. firmus* have been reported to cause mortality of *M. incognita* (Mendoza et al., 2008); however, the short duration of this experiment may not have given the bacterium time to produce a sufficient amount of secondary metabolites to affect the nematodes.

The movement of fluopyram was greater in sandy soil as a soil-applied treatment with reductions in movement in a sandy loam soil or as a seed-applied treatment. This study supports the importance of the soil texture and physical properties of nematicides when investigating nematicide efficacy and provides a better understanding as to the downward movement of seed- and soil-applied fluopyram in sandy soil.
